# How can an in vitro incompatibility of Trichoderma-based products and herbicides impact the parasitism and control of white mold (*Sclerotinia sclerotiorum* (Lib.) De Bary)?

**DOI:** 10.1007/s44297-024-00024-1

**Published:** 2024-04-09

**Authors:** Lindomar Canuto da Silva, Amanda Flausino de Faria, Rafaela Araújo Guimarães, Muhammad Siddique Afridi, Flavio Henrique Vasconcelos de Medeiros, Fernanda Carvalho Lopes de Medeiros

**Affiliations:** 1 Department of Agriculture, Universidade Federal de Lavras, Campus Universitário, CP, MG, 3037, Lavras, 37203-202 Brazil; 2 Department of Plant Pathology, Universidade Federal de Lavras, Campus Universitário, CP, MG, 3037, Lavras, 37203-202 Brazil

**Keywords:** Glycine max, Compatibility, Glyphosate, *Trichoderma* spp, *S. sclerotiorum*, White mold

## Abstract

The integration of management methods for both diseases and weeds depends on the compatibility between the tools. Biological control represents an important strategy to cope with the integrated management of white mold (*Sclerotinia sclerotiorum*) through parasitism of sclerotia. However, its application in the field is more cost-effective if combined with the herbicide in a tank mix, as long as the products are compatible. Therefore, we aimed at (i) evaluating two compatibility test methodologies (constant exposure and different times) and (ii) two soybean crop seasons to infer the compatibility of Trichoderma-based products. In vitro bioassays were performed to assess the compatibility between herbicides (Haloxifope-p- methyl, Glyphosate N-ammonium salt, Fluasifope-p-butyl, Fomesafem, Chlorimuron ethyl and Imazapyc + Imazapyr) and two biocontrol agents (*Trichoderma asperellum* and *Trichoderma harzianum*). Thus, the recommended spray volume for each herbicide was added to the PDA culture medium (Potato-Dextrose-Agar) and then deposited in the center of the plate a disc of mycelium from each antagonist isolate (constant exposure). The tests with time of exposure were marked at times 0, 2, 4, 8 and 16 h (simulating tank mixing) and at the spp of each time were plated on PDA medium. For both tests, the mycelial growth and conidiogenesis of *Trichoderma* spp. were observed. The combination of herbicide and biocontrol was also tested in the field and sclerotia parasitism, white mold incidence and plant yield were assessed in two field trials. The constant exposure of the antagonists to herbicides revealed that no herbicide was compatible with the *T. asperellum* or *T. harzianum*. While in test, exposure time exhibited compatibility with either *T. asperellum* or *T. harzianum*, within a period of 2 to 8 h. Conclusively, the integration of biocontrol agents with the herbicide imazapique + imazapyr exhibited significant reductions in white mold disease incidence and conidia germination, along with effective parasitism of *S. sclerotiorum* and even the least compatible herbicide (glyphosate) resulted in significant reduction in the disease incidence and sustained yield when compared to the untreated control. Therefore, the integration of the biocontrol agent for white mold should always be considered, and the tank mixing of it with the herbicide represents a cost-effective alternative for the grower.

**Supplementary Information:**

The online version contains supplementary material available at 10.1007/s44297-024-00024-1.

## Introduction

White mold, caused by *Sclerotinia sclerotiorum* (Lib.) De Bary, is a devastating soybean disease that can cause yield reductions of up to 60% worldwide [1]. This pathogen forms overwintering structures known as sclerotia, which can either germinate directly to produce mycelium and infect nearby plants or develop into apothecia, releasing ascospores that have the potential to infect a multitude of plants within a radius of 100 m [2]. The infection caused by the pathogen is of critical importance during the flowering stage of most dicot plants [3, 4]. This is because the ascospores rely on the availability of nutrients provided by flowers in order to initiate the infection and facilitate the development of the disease [2]. Hence, the application of chemical fungicides during the flowering stage has been suggested as the most viable approach for safeguarding plants. However, it does not provide comprehensive plant protection [5].

Therefore, approaches for managing diseases have been suggested: genetic (breeding), chemical, biological and cultural control. Breeding for resistance to *S. sclerotiorum* poses challenges due to the polygenic nature of resistance [6]. The feasibility of implementing control measures through crop rotations is limited by the long-term presence of survival structures (sclerotia) in the soil and the broad spectrum of host plants susceptible to *S. sclerotinia* [7]. Moreover, cover crops have also been suggested as an effective strategy for disease management [8]. However, the most commonly used strategy worldwide relies on chemical fungicide upon flowering [9].

Biological control strategies have emerged as among the most effective approaches into large-scale disease management over the past decade [10].

Biological control of white mold aims to reduce the initial source of inoculum (sclerotia) [11]. *Trichoderma* species, employed as biological control agents, are strategically utilized to target the primary source of inoculum (sclerotia) [12]. These species parasitize sclerotia, resulting in a reduction in apothecia formation and subsequently decreased release of ascospores, thereby leading to a reduced infection rate. The majority of [13] biocontrol products feature *T. harzianum* and *T. asperellum*, which are applied during the early vegetative stages (V2 and V4) of soybean development [14, 15].

Soybean is the main crop in Brazil and its cultivation is of great importance, both for the country’s economy and for the global market [16]. Soybeans are a cornerstone of Brazil’s economy and global market, with the country ranking as the second-largest producer and exporter. During 2019–2020 harvest, the soybean complex saw a notable surge, producing over 33 million tons—an 80% increase compared to the last decade. This output represents 27% of the national soybean production, which is valued at almost USD 9 billion in exports, equivalent to 25 million tons of soybeans [16–19]. In Brazil, the utilization of both selective and non-selective herbicides is crucial in the management of soybean cultivation to effectively control weeds. In Brazil, the management of soybean cultivation using selective and non-selective herbicides has been characterized by the extensive adoption of glyphosate-resistant soybean cultivars and a rise in the utilization of alternative herbicides to combat glyphosate-resistant weeds. The total area of soybean crops treated with herbicides increased by 10%, from 154.7 to 170.5 million hectares, during the years of 2017/2018 and 2019/2020. In 2019, the herbicides most commonly utilized in Brazil were glyphosate, 2,4-D, atrazine, paraquat, and diuron, accounting for 62%, 15%, 7%, and 3% of the overall herbicide consumption, respectively [20–22].

Integrated Pest Management (IPM) is a holistic and sustainable approach to pest and disease management in agriculture, that combines various strategies to minimize the impact of pests while ensuring the health and productivity of crops. Biological control products are integrated into pest management, alongside chemical agents such as fungicides, insecticides, and herbicides, within the framework of IPM [23, 24]. The routine and common practice in the field involves mixing biological control products with pesticides in the same tank, which can result in cost savings for farm operations [25, 26].

In the context of white mold disease management, the integration of Trichoderma-based products into herbicide mixtures is a prevalent and established practice. However, simultaneous application of biocontrol sprays for disease management and post-emergence herbicides for weed control may potentially impact the performance of *Trichoderma* [27]. Although, the available information on this matter presents a degree of contradiction. However, Ramirez et al. (2021) [28] suggested that certain herbicides, such as glyphosate, may reduce the viability of *Trichoderma* sp. while still allowing it to effectively display its biocontrol activity.

The ability of an incompatible interaction observed in vitro to translate into sustained product performance in the field may be attributed to various factors, with exposure time being a crucial consideration. In the field, the biocontrol agent is typically exposed to the herbicide for a relatively brief period of 2–4 h during the tank mixing process. In contrast, compatibility assays involve amending the growth medium with the herbicide at the same rate employed in the field and subsequently assessing mycelial growth or conidia germination over a specified duration [24].

The aim of this research study was (1) to evaluate the impact of various herbicides (haloxyfope-P-methyl, glyphosate salt N-ammonium, fluasifope-p-butyl, flexfomesafen, cllorimuron ethyl and imazapyc + imazapyr) on the growth of *Sclerotinia sclerotiorum*, including the number and weight of sclerotia. Additionally, (2) this study aimed to assess the compatibility of these herbicides with *Trichoderma asperellum* and *Trichoderma harzianum*, as well as their compatibility at different exposure times (0, 2, 4, 8 and 16 h) with these fungi. Finally, (3) the study aimed to evaluate the impact of mixtures of these herbicides with *Trichoderma* sp. on the incidence of white mold and yield.

## Materials and methods

### Obtaining and multiplication of *Sclerotinia sclerotiorum*

The *S. sclerotiorum* strain used in these experiments was obtained from the Ingaí municipality (21° 24′ 03" S 44° 55′ 01" O) in the southern part of 113.Minas Gerais (Brazil) at 971 m of altitude. The region has a tropical climate at altitude and the isolate was obtained from symptomatic soybean plants, as described by Alves et al. (2021) [29].The sclerotia were subjected to a comprehensive surface sterilization protocol, beginning with a 30-s immersion in 70% ethanol, followed by a 2-min exposure to a 2% sodium hypochlorite solution. Subsequently, they were rinsed three times with distilled water. To propagate the isolate and generate the myceliogenic mass, a mycelial disc was aseptically placed onto a potato dextrose agar (PDA) medium. This culture was then incubated under controlled conditions at 25 °C with a photoperiod of 12 h, spanning a duration of 7 days [7].

### Compatibility assay

The assessment of compatibility between biocontrol agents and herbicides was performed using an in vitro assay at the Laboratory of Biological Control, Department of Phytopathology, Federal University of Lavras (UFLA), MG, Brazil. The field experiments were conducted over two consecutive crop seasons (2019/2020 and 2020/2021) within soybean crop fields. The objective of these experiments was to assess the efficacy of biological control agents and herbicide combinations in managing white mold disease.

### The sensitivity bioassay of *Sclerotinia sclerotiorum* to different herbicides

The sensitivities of *S. sclerotiourm* to herbicides [Verdict®R (haloxifope-P-methyl 12.46%), Roundup Original Di® (glyphosate salt N-ammonium 44.5%), Fusilade® 250 EW (fluasifope-p-butyl 25%), Flex® (flexfomesafen 25%), Classic® (cllorimuron ethyl 25%) and Amplexus® (imazapyc 52.5% + imazapyr 17.5%)] were assessed at the recommended field dose with slight modifications proposed by (AGROFIT, 2021; Alves et al., 1998). A total volume of 200 mL of PDA medium was precisely prepared in accordance with the recommended field application protocol. Herbicides were applied at the appropriate amount per hectare, and afterwards, 15 mL of this herbicidal mixture was applied to each individual Petri dish. A mycelium plug measuring 5 mm in diameter was precisely put in the center of each Petri plate. Subsequently, the Petri dishes were incubated at a temperature of 25 °C with a photoperiod of 12 h for a total duration of 15 days. The control group exclusively included the *S. sclerotiorum* mycelium plugs on BDA medium, without the incorporation of any herbicide. The mycelium plug of *S. sclerotiorum* was cultivated on PDA and considered as the control. The herbicides dose used was according to Table S1, considering a spray volume of 200 L ha^−1^.

### Sclerotia and mycelial growth assessment of *S. sclerotiorum*

The effect of herbicides on the pathogen *S. sclerotiorum* was assessed by measuring the diameter and mycelial growth, as well as the number and weight of sclerotia formed during the herbicide-pathogen interaction. To assess the mycelial growth, the horizontal and vertical diameters were measured using a digital caliper. The measurements were conducted at 24-h intervals until the control treatment completely covered the surface of the Petri dish. Subsequently, the data obtained were employed to calculate the Mycelial Growth Rate Index (MGRI), following the equation elucidated by [24]. After a period of 15 days of cultivation under controlled conditions, the melanized plates were utilized to collect, count, and weigh the produced sclerotia. The weight of the sclerotia was measured using an analytical scale, shown in grams.

1 $$\mathrm{MGRI }=\frac{\sum \left({\mathrm{D}}-{\mathrm{Da}}\right)}{{\mathrm{N}}}$$

Where: MGRI = mycelial growth rate index; D = current average colony diameter; Da = average diameter of the previous day’s colony; and N = number of days after inoculation.

### The sensitivity bioassay of *Trichoderma spp.* to different herbicides

The effect of herbicides on biological products based on *T. harzianum* and *T. asperellum* were evaluated using measurements of mycelial growth, conidia production and inhibition of antagonist mycelial growth in comparison to the control treatment. In this experiment, continuous exposure of *T. harzianum* and *T. asperellum* to the herbicides was investigated using the Petri dishes methodology, containing the PDA culture medium with herbicides as mentioned above. However, the methodology of constant exposure of antagonists to the herbicide does not represent accurately and does not reflect practical applications practices in field conditions employed by producers. In order to simulate an application in which the products are mixed in a tank and then sprayed, an experiment using another methodology considering different exposure times (0, 2, 4, 8 and 16 h) of antagonists to herbicides, simulating the mixing in tanks that possibly occurs in field conditions was carried out.

### Assessment of the constant exposure of biological control agents to herbicides

In this experiment, the herbicides were added to 200 mL of PDA culture medium that was in a liquid state at a temperature of about 45 ºC. The culture medium had been autoclaved previously, and the application of the herbicides followed the recommended spray volume and dosage per hectare. Subsequently, a volume of 15 mL of the mixture comprising of PDA and herbicides was poured within a Petri dish with a diameter of 90 mm. After the solidification of the medium, a suspension containing biological products was inoculated onto a filter paper disc with a diameter of 5 mm, which was placed in the center of a Petri dish. This process was performed using an automatic pipette. The Petri dishes were then sealed with plastic film and stored in a BOD (biological oxygen demand) chamber at a temperature of 25 °C, a relative humidity of 70 ± 10%, and a photoperiod of 12 h for 7 days. In the control treatments, the Petri dishes were supplemented with the PDA culture medium without the addition of the herbicide. Furthermore, the biological products were added to the Petri dishes following the aforementioned protocol**.** The dose of herbicides and biological products were used in accordance with Table S1, considering a spray volume of 200 L ha^−1^.

### Assessment of the exposure of biological control agents to herbicides at different exposure times

In this experiment, a different methodology was employed from the one described above. The herbicides and biological products were incorporated into 200 mL of autoclaved distilled water in a beaker according to the designed treatments. This was performed in accordance with the recommended spray volume of 200 L ha^−1^ and the prescribed dosage specified in the package leaflet per hectare, as mentioned in Table 2. After mixing and homogenizing the products, a 40 mL aliquot was extracted from each beaker and transferred into a falcon tube. Subsequently, the falcon tube was immersed in an ultrasound bath for a period of 3 min, and then subjected to constant automatic agitation at 150 rpm.

At each exposure time, a 5 mL aliquot was extracted and subjected to centrifugation to facilitate the separation of *Trichoderma* conidia from the herbicide present in the solution. This 5 mL aliquot was homogenized by vortexing for 1 min, followed by the extraction of a 1 mL aliquot and that was transferred into a microtube. This procedure was repeated 4 times for each treatment in the referred exposure time, with each microtube representing a repetition. Microtubes were centrifuged at 10,000 rpm for 4 min to form a pellet containing the fungus conidia. This pellet was resuspended in distilled water and plated.

Plating was performed in 90 mm Petri dishes containing solidified PDA culture medium, with 20 μL of the pellet solution pipetted and inoculated on top of a 5 mm diameter filter paper disc inserted in the middle of the Petri dish.

Plates were sealed with plastic film and kept in the B.O.D. at 25 ± 1 °C temperature, 70 ± 10% relative humidity and 12 h of photoperiod for 7 days. For the control treatments, only the biological products were left untreated.

### Mycelial growth assessment of biocontrol agents

To assess mycelial growth and diameter produced by the fungus was measured with the aid of a digital caliper in two transversal directions, determining the average growth diameter. These measurements were taken every 24 h until the first contact between one of the fungal colonies and the plats edge was observed. These data were used to calculate the mycelial growth rate index (MGRI), according to the formula described by [24] (2022), (Eq. 1). Based on the results of mycelial growth, the percentage of mycelial growth inhibition (PMGI) in relation to control was calculated, according to the methodology proposed by [30] (2013) (Eq. 2).

2 $$\mathrm{PMGI }= \left(\frac{\mathrm{\varnothing\ Control\ treatment\ }-\mathrm{ \varnothing\ of\ the\ treatment}}{\mathrm{\varnothing\ Control\ treatment}}\right)\times 100$$

Where: PMGI = percentage of mycelial growth inhibition; ∅ = average colony diameter; At 7 days, three 5 mm diameter plugs from the *Trichoderma* colony were removed from each Petri dish for the conidia quantification. Each plug was placed in a 50 mL-conical tube with 5 mL of sterile saline + Tween 80 (0.1%), being vortexed for about two minutes, until the conidia were scrapped from the surface of the mycelial plug. After removing the conidia, counting was performed with the optical microscope at 200x magnification in a Neubauer chamber, using dilutions of the suspension when necessary.

### In-vivo experiments

#### Assessing the in Vivo efficacy of biological products combined with herbicides for *Sclerotinia sclerotiorum* control in 2019/2020 crop season

The experiment comprised two consecutive soybean production cycles within the time frame of the 2019/2020 and 2020/2021 seasons. The study was conducted during the 2019/2020 season at Papagaio Farm, an agricultural region located inside the Municipality of Luminárias, MG. The geographical coordinates of the region are 21°29′10" S latitude and 44°58′15" W longitude, with an elevation of 948 m. The research was conducted from November 11th, 2019 to March 25th, 2020. The soybean cultivar used in the 19/20 season was Desafio RR- 8473 RSF (Brasmax), with an indeterminate growth habit and a relative maturation group of 7.4, sown with 60 cm spacing between rows and 15 seeds per linear meter, with a stand end of 250 thousand plants per hectare. Fertilization was carried out according to the soil analysis, using 150 kg ha^−1^ of potassium chloride applied to haul in the pre-planting and 200 kg ha^−1^ of monoammonium phosphate (MAP) applied in the planting furrow. The seed treatment was carried out with the fungicide /insecticide Standak Top in the dose of 200 mL for 100 kg of seeds. In addition, inoculation was performed with 7 doses of *Bradyrhizobium japonicum* and 2 doses of *Azospirillum brasilense*.

During the desiccation process, Cypermethrin from Nortox was applied at a rate of 200 mL ha^−1^, along with the application of 1.5 kg ha^−1^ of boric acid through a desiccation syrup. This was done to provide essential boron to the soil. Additionally, the control of Asian rust was managed by applying the FOX® fungicide in combination with Unizeb Gold during the R1 and R3 growth stages.

#### Assessing the in Vivo efficacy of biological products combined with herbicides for *Sclerotinia sclerotiorum* control in 2020–2021 crop season

The experiment was carried out throughout the 2020/2021 season in the agricultural setting of Santa Maria Farm, situated in the municipality of Conceição do Rio Verde, MG. The coordinates of the location are 21°53′26″ S latitude and 45°06′37″ W longitude, with an altitude of 898 m. The current phase of the study was conducted between October 22nd, 2020, and February 23rd, 2021.

In the 2020/21 season, the soybean cultivar Lança 58i60 RSF IPRO (Brasmax) was used, with an indeterminate growth habit and a relative maturation group of 5.8, sown with 45 cm spacing between rows and 13.5 seeds per linear meter, totaling a population of 300 thousand plants per hectare. Fertilization was carried out according to the soil analysis, with 200 kg ha^−1^ of potassium chloride applied to haul in the pre-planting and 200 kg ha^−1^ of monoammonium phosphate (MAP) applied in the planting furrow. The seed treatment was carried out with the fungicide / insecticide Standak Top in the dose of 200 mL for 100 kg of seeds. Inoculation was performed with 8 doses of *Bradyrhizobium* japonicum and 2 doses of *Azospirillum brasilense*. The desiccation of the area was carried out 15 days before soybean sowing, using the herbicides Crucial, Aminol 806 and Classic at the doses of 3.0 L ha^−1^, 1.5 L ha^−1^ and 80 g ha^−1^, respectively. For desiccation, it was also performed with the application of Cypermethrin Nortox in the dose of 200 mL ha^−1^, in addition to the application of 2.0 kg ha^−1^ of boric acid via desiccation syrup. The control of Asian rust was carried out by applying the FOX® fungicide combined with Unizeb Gold in the R1 and R3 growth stages.

### Disease assessment evaluation

To assess disease control, we conducted incidence evaluations for white mold at growth stages R5.1 and R5.4. These assessments involved 40 plants sampled from the two central rows within each experimental plot. At the R8 stage of plant development, all plants from the relevant plots were harvested to ascertain productivity, measured in kilograms per hectare (kg ha^−1^). The calculation of grain productivity was based on the weight of grains from each useful plot, converted to kg ha^−1^. Grain moisture content was adjusted to a 13% wet basis during these calculations.

### Data analysis

The experimental design used was randomized blocks (DRB) in a factorial scheme as (7 × 2) + 2, resulting in seven treatments (*T. asperellum* mix with six different herbicide + only *T. asperellum* = 7) x (*T. harzianum* mix with six different herbicide + only *T. harzianum* = 7) equal 7 × 2 more two controls (only water and only fungicide), with 4 replications for a total of 64 experimental plots. Each treatment was replicated four times. Each experimental plot was represented by eight lines of 5.0 m in length, the useful area of each plot being the four central lines, excluding 0.5 m at each end of the line. The products and the respective doses used to evaluate the efficiency of the control of white mold are found in Table S1. The treatments were applied in vegetative stages V2 and V4, and in the first application only the application of the biocontrol agent was performed, while in the second application the biocontrol agents were applied combined with the herbicides in the tank mixture. The doses were diluted in a volume of 200 L ha^−1^ and the sprays were carried out with the aid of a CO_2_ pressurized sprayer coupled to a plastic 2L bottle, with a bar consisting of 4 tips of the type XR110.02 spaced 0.5 m apart, under a pressure of 2 atm (202.65 kPa). The concentrations of the used products are presented in Table S1.

The results obtained were evaluated by the tests of normality and homogeneity test and subsequently submitted to analysis of variance based on the design adopted for p less than or equal to 5% of probability, by the F test, using the statistical program R Studio. When significant differences were detected, the means were compared using the Tukey’s test for in vitro assay and in field trials.

## Results

### Evaluating the impact of herbicides on propagule abundance and mycelial growth rate index of biocontrol agents *T. harzianum* and *T. asperellum*

**Fig. 1:**
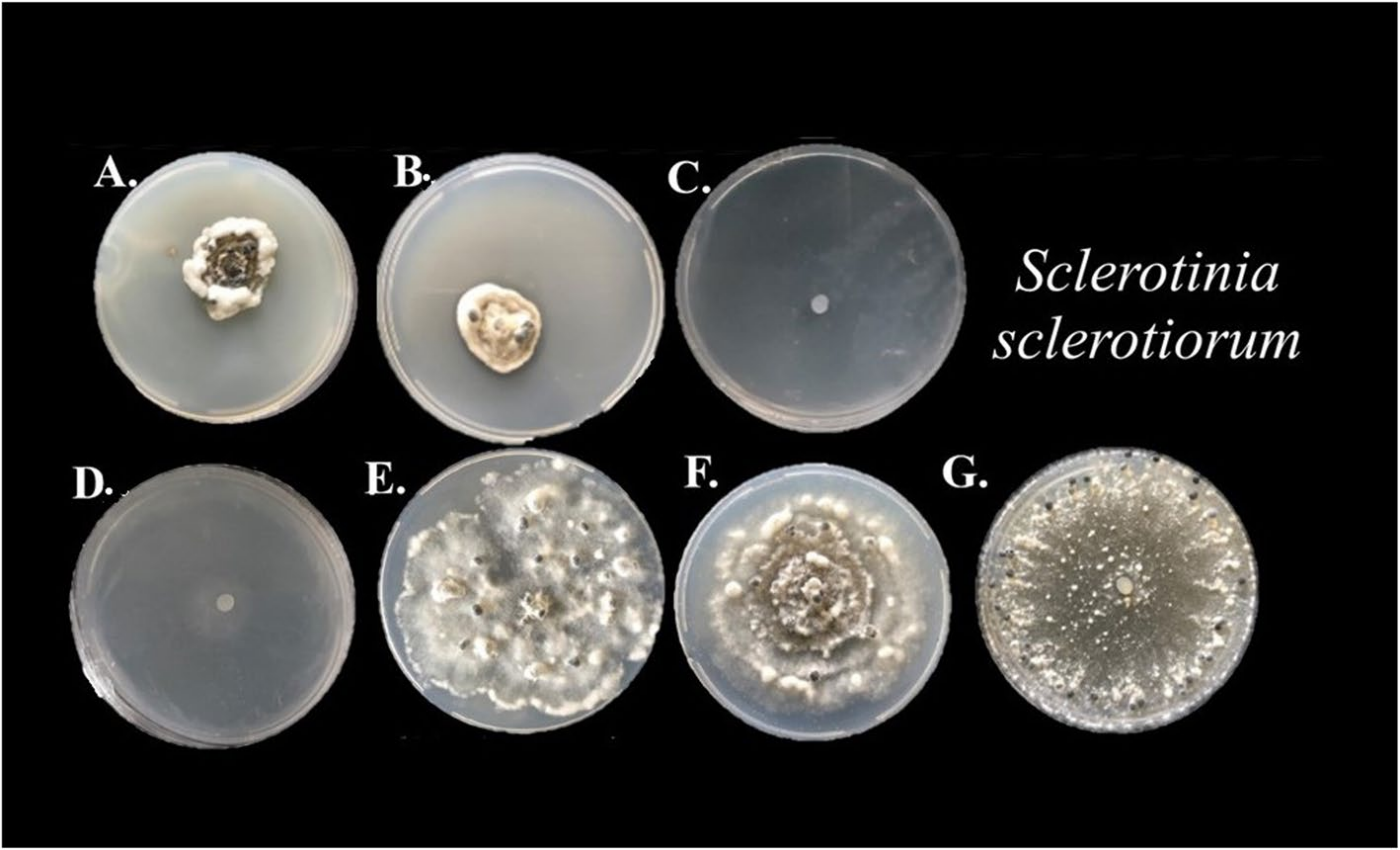
Number of sclerotia produced by *Sclerotinia sclerotiorum* in potato-dextrose-agar culture medium with different herbicides at 15 days of incubation. **A** Fluasifop-p-butyl; **B** Haloxyfop-p-methyl; **C** Glyphosate; **D** Chlorimuron ethyl; **E** EImazapyc + imazapyr; **F** Fomesafen; **G** water. Mean followed by the same letter are not significantly different (*p* < 0.05) according to the Tukey’s HSD test

**Fig. 2:**
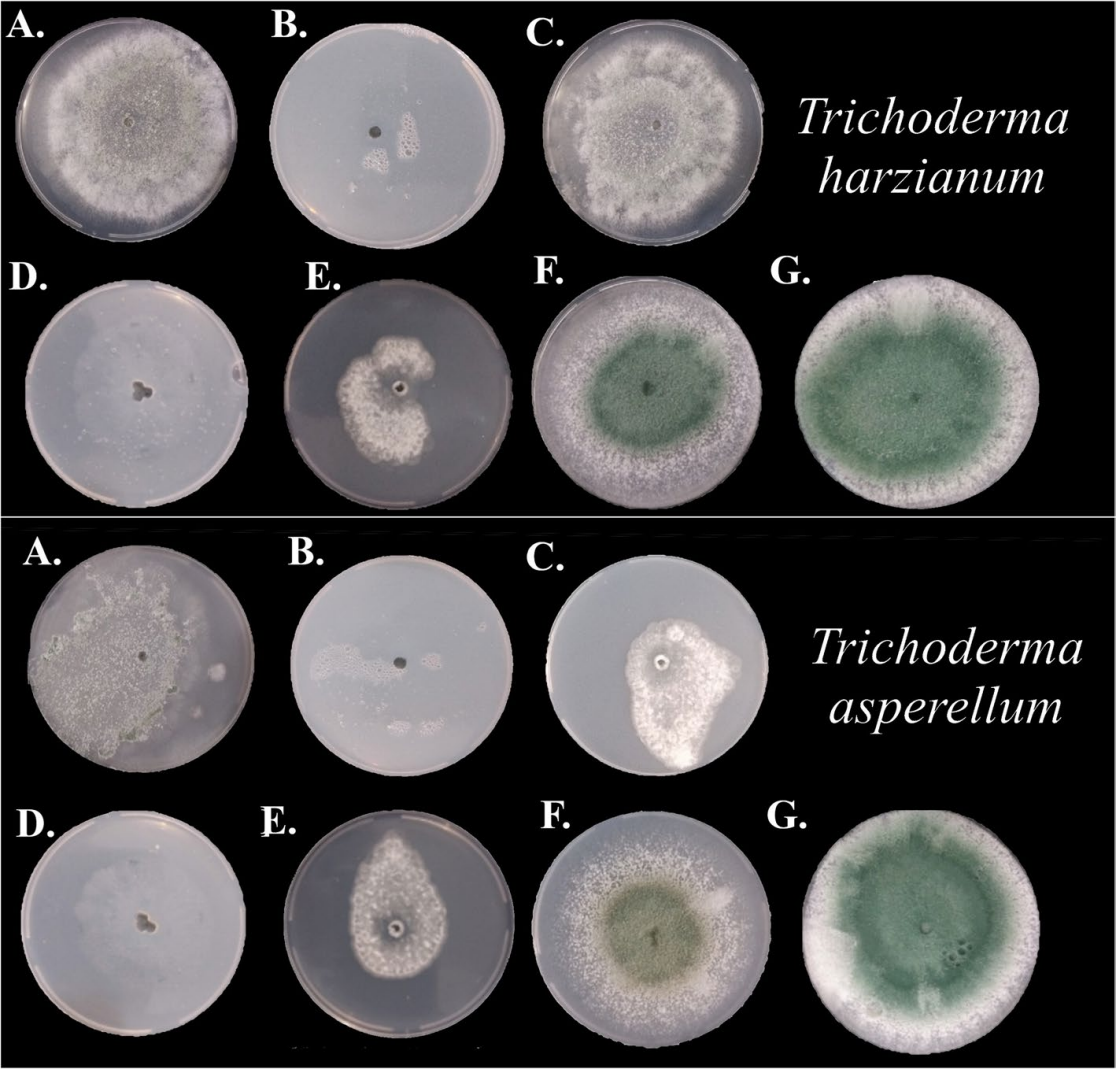
Mycelial growth of *Trichoderma harzianum* and *Trichoderma asperellum* in potato-dextrose-agar culture medium with different herbicides at 15 days of incubation. **A** Fluasifop-p-butyl; **B** Haloxyfop-p-methyl; **C** Glyphosate; **D** Chlorimuron ethyl; **E** EImazapyc + imazapyr; **F** Fomesafen; **G** water. Mean followed by the same letter are not significantly different (*p* < 0.05) according to the Tukey’s HSD test

Either an initial compatibility test considering full exposure to the different herbicides in the Petri dish of the pathogen (Fig. 1) or the biocontrol agent (Fig. 2) showed deleterious effects on the pathogen (Table 1) and the number of propagules and mycelial growth rate index of the biocontrol agents (Table 2). Actually, it reduced conidia production by approximately 60% compared to the group exposed for 0 h (Table 3). The MGRI parameter showed significant differences between the exposure times, but the difference between the time between 0 and 16 h was 1.51 mm day^−1^ (Table 3). The antagonists, *T. asperellum* and *T. harzianum*, had more detrimental effects from the herbicide glyphosate. The other herbicides had milder detrimental effects, e.g., Fomesafen reduced the growth of *T. asperellum* when compared to the control without herbicide addition by 27.80% within 16 h of exposure and caused a reduction of approximately 80% in the fungal sporulation (Table 3).

**Table 1:** Effect of herbicides on the weight of sclerotia (WS) and percentage of inhibition of mycelial growth (PIMG) of *Sclerotinia sclerotiorum*. UFLA, Lavras/MG, 2021. Either delete this information or add it into the statement mean mention the table number

Treatment (Herbicides)	WS (g)	PIMG (%)
Control	0.18^ab^	0.00^d^
Haloxyfop-p-methyl	0,077^c^	89,75^b^
Glyphosate N Di-ammonium Salt	0,000^d^	100,00^a^
Fluasifop-p-butyl	0,026^cd^	81,05^c^
Fomesafem	0,171^b^	85,19^bc^
Ethyl Chlorimuron	0,000^d^	81,37^c^
Imazapique + Imazapir	0,243^a^	79,68^c^

Means followed by the same letter do not differ from each other using the Tukey test, at 5% probability

**Table 2:** Effect of herbicides on two biocontrol agents (*Trichoderma harzianum* and *T. asperellum*) and the plant pathogen (*Sclerotinia sclerotiorum*) measured by the sporulation number of propagules (NP), Mycelial growth rate index (MGRI) of fungi in mm day^−1^. Means followed by the same letter do not differ from each other using the Tukey test, at 5% probability

Herbicides	**NP**			**MGRI (mm day**^**−1**^**)**		
	*T. harzianum*	*T. asperellum*	*S. sclerotiorum*	*T. harzianum*	*T.asperellum*	*S. sclerotiorum*
Control	14.4^*a^	12.1^a^	22.2^a^	22.5^*a^	18.0^a^	26.7^a^
Haloxyfop-p-methyl	2.7^c^	1.0^b^	2.0^cd^	9.9^*c^	2.8^d^	2.7^c^
Glyphosate N-ammonium salt	0.0^d^	0.0^c^	0.0^d^	0.0^d^	0.0^e^	0.00^d^
Fluasifop-p-butyl	4.9^*b^	1.4^b^	3.5^c^	16.2^*b^	10.2^b^	5.1^b^
Fomesafen	4.2^*b^	1.3^b^	11.5^b^	16.7^*c^	6.1^c^	3.9^bc^
Chlorimuron ethyl	3.4^*b^	1.6^b^	0.0 d	11.1^*a^	6.1^c^	5.00^b^
Imazapyc + Imazapyr	4.1^*bc^	1.7^b^	17.5^ab^	22.4^*a^	17.8^a^	5.4^b^

**Table 3:** Mycelial growth rate index (MGRI) in mm day^−1^ and number of conidia mL^−1^ (NC) of biocontrol agents (*Trichoderma harzianum* IBLF 006 and *Trichoderma asperellum* BV10) caused by the herbicides amendment at different times of exposure (0, 2, 4, 8 and 16 h) at the concentrations used for plant spray. Means followed by the same lowercase letter in the row and capital letter in the column do not differ from each other, according to the Tukey test at 5% probability. Capital letters refer to biological control agents, while lowercase letters refer to herbicide

Time (Hours)	Control	T*. harzianum* MGRI (mm h^−1^)	*T. asperellum* MGRI (mm h^−1^)	T*. harzianum* NC (conidia mL^−1^)	*T. asperellum* NC (conidia mL^−1^)
0		18.0*a Aa	18.0 Aa	12.0 Aa	9.8 Aa
2		18.0*a Aa	18.0 Aa	11.4 *Aa	8.6 BaB
4		18.0*a Aa	18.0 Aa	11.2 *Aa	5.1 Bbc
8		18.0*a Aa	15.0 Bb	7.5 *Ab	3.9 Bc
16		18.0*a Aa	15.0 Bb	7.4 *Ab	3.5 Bc
	**Haloxyfop-p-methyl**				
0		17.5a Aa	16.8 Ba	16.5 *Aa	8.4 Ba
2		17.3a Aa	16.6 Ba	15.3 *Aa	7.2 Bab
4		17.1a Aa	14.8 Bb	9.9 *Ab	5.3 Bab
8		15.7b Ab	14.7 Bb	10.9 *Ab	3.9 Bbc
16		13.3*c Ac	8.8 Bc	3.0 Ac	1.3 Ac
	**Glyphosate N-ammonium salt**				
0		12.9 Aa	12.3 Ba	8.3 *Aa	4.8 Ba
2		0.0 Ab	0.0 Ab	7.1*Aab	0.0 Bb
4		0.0 Ab	0.0 Ab	4.0 *Ab	0.0 Bb
8		0.0 Ab	0.0 Ab	0.0 *Ac	0.0 Ab
16		0.0 Ab	0.0 Ab	0.0 Ac	0.0 Ab
	**Fluasifop-p-butyl**				
0		17.6a Aa	17.5 Aa	15.9* Aa	7.2 Ba
2		17.5ab Aab	17.3 Aa	14.7* Aa	6.0 Ba
4		17.4*ab	15.8 Bb	12.9* Aa	6.2 Ba
8		16.7*bc Abc	14.3 Bc	9.5* Ab	4.9 Bab
16		15.9*c Ac	10.9 Bd	5.5* Ac	1.4 Bb
	**Fomesafen**				
0		17.7a Aa	17.3 Aa	10.8* Aa	7.1 Ba
2		17.4ab Aab	17.1 Aa	9.6* Aab	5.9 Ba
4		16.7*bc Abc	14.5 Bb	7.1* Abc	3.9 Bab
8		16.4*c Ac	14.6 Bb	7.1* Abc	3.9 Bab
16		16.1*c	10.8 Bc	4.3*Ac	1.5 Bb
	**Chlorimuron ethyl**				
0		17.3*a Aa	16.4 Ba	12.7* Aa	8.5Aa
2		17.1*ab Aab	16.2 Ba	11.5* Aa	7.3Aab
4		16.8*ab Aab	15.9 Ba	5.4 Abc	3.5Bc
8		16.5*b Ab	14.5 Bb	7.1* Ab	3.8Bbc
16		15.7* Ac	10.4 Bc	3.5 Ac	1.8Ac
	**Imazapyc + Imazapyr**				
0		17.6aAa	17.5Aa	10.6Aa	8.5Aa
2		17.5a Aab	17.3Aa	9.4Aa	7.3BaB
4		17.2*a Aab	14.8Bb	9.6* Aa	3.5Bbc
8		16.8*b Ab	14.0Bb	8.6* Aa	3.8Bc
16		15.8*c Ac*	11.4Bc	4.2 Ab	1.8Bc

### Effect of herbicides on the number of sclerotia produced (NS), weight of sclerotia (WS) and percentage of inhibition of mycelial growth (PIMG) of *Sclerotinia sclerotiorum*

A first group with total inhibition (glyphosate N-ammonium and chlorimuron ethyl), a high-impacting group with 84–90% (fluasifop-p-butyl and haloxyfop-p-methyl), an intermediate group with 48% (fomesagen) and an herbicide that had no significant effect on sclerotia formation (imazapyc + imazapyr) were found in this study (Fig. 2, Table 1).

### In vitro evaluating the mycelial growth of *Trichoderma harzianum* and *Trichoderma asperellum in* potato-dextrose-agar culture medium with different herbicides after 15 days of incubation

For both variables and all three fungi, glyphosate N-ammonium did not allow the mycelial growth or sporulation. In regard to propagule quantity, *T. harzianum* exhibited a consistently higher rate of sporulation compared to *T. asperellum*, even for the untreated control (Fig. 2, Table 2). After exposure of the different herbicides to biocontrol agents, the sporulation *T. harzianum* was reduced by 71–100% and *T. harzianum* was reduced the sporulation by 86–100% compared to the control. Glyphosate N-ammonium salt, when exposed to *T. harzianum* resulted in the highest reduction of sporulation. With respect to the herbicides tested, it was observed that *T. harzianum* exhibited reduced sensitivity, particularly in comparison to haloxyfop-p-methyl and glyphosate N-ammonium salt. Interestingly, the sensitivity of *T. harzianum* was notably lower when exposed to Imazapyc + imazapyr (Table 2). This was the considered variable for the number of propagules and a pattern of sensitivity was observed different from *Trichoderma* spp. Nevertheless, despite being exposed for a duration of 16 h, both fungi exhibited continued mycelial development and sporulation. Remarkably, the growth of both fungi was found to be hindered by Glyphosate N-ammonium salt after a period of five days, during which the mycelial growth was assessed. This duration was consistent across all tested products and allowed sufficient time for the conidia, which had not been subjected to the herbicide, to reach the periphery of the plate. The measurement of sporulation rate was conducted seven days following the transfer of conidia. At this time point, it was feasible to examine both the growth and sporulation of *T. harzianum*.

### In vitro compatibility test between herbicides and biological products: exposure of biological control agents to herbicides at different exposure times

The fungal viability exhibited variation based on the duration of exposure to the herbicides. The effects of the herbicides were compared with those of *Trichoderma* species at different exposure times (Table 3).

After a prolonged period of exposure (8 h), the mycelial growth index (MGRI) and sporulation of haloxyfop-p-methyl have been observed to be significantly reduced (Table 3). *T. harzianum* reduced mycelial growth up to 12–25% followed by 41.3 T*. asperellum,* mycelial growth reduction up to 41.3% compared to control (Table 3). A reduction of up to 53% in sporulation was found for *Trichoderma harzianum* (Table 3).

In 16 h, the two species of *Trichoderma* presented the lowest MGRI, in addition to a reduction in the production of conidia of 65% for *T. harzianum* and an 80% reduction for *T. asperellum,* (Table 3).

Combining antagonists with Fluasifope-p-butyl herbicide at tested doses maintains high efficacy for up to 8 h, suggesting a feasible alternative to the common practice of immediate spraying after tank mix preparation among growers.

The herbicide contains Fomesafen at the longest exposure time (16 h) caused a 10.82% decrease in the growth of the *T. harzianum* fungus compared to the control group without herbicides (Table 3).

Chlorimuron ethyl, the active ingredient, significantly reduced sporulation by approximately 40% after 4 h of exposure compared to the initial time for both fungi, and in the PMGI evaluation, it led to a greater reduction in *T. asperellum’*s growth by 30.83% and statistically significant mycelial growth inhibition ranging from 3.57% to 12.87% for *T. harzianum* across exposure times.

Regarding the MGRI, there were significant differences in which the time of 0 h presented the best result with 17.65 mm day^−1^ (Table 3).

The mycelial growth of *T. harzianum* showed no statistically significant differences in a mixture with Imazapyc and Imazapyr herbicide from 0 to 8 h, ranging from 1.91% to 6.44%. However, at 16 h, a significant reduction of 12.18% was observed, which was less than the growth inhibition caused to *T. asperellum* (24.14%) by the same herbicide (Table 3).

### Two-year in Vivo assessment of biological product efficacy in synergy with herbicides for *Sclerotinia sclerotiorum* control

**Fig. 3:**
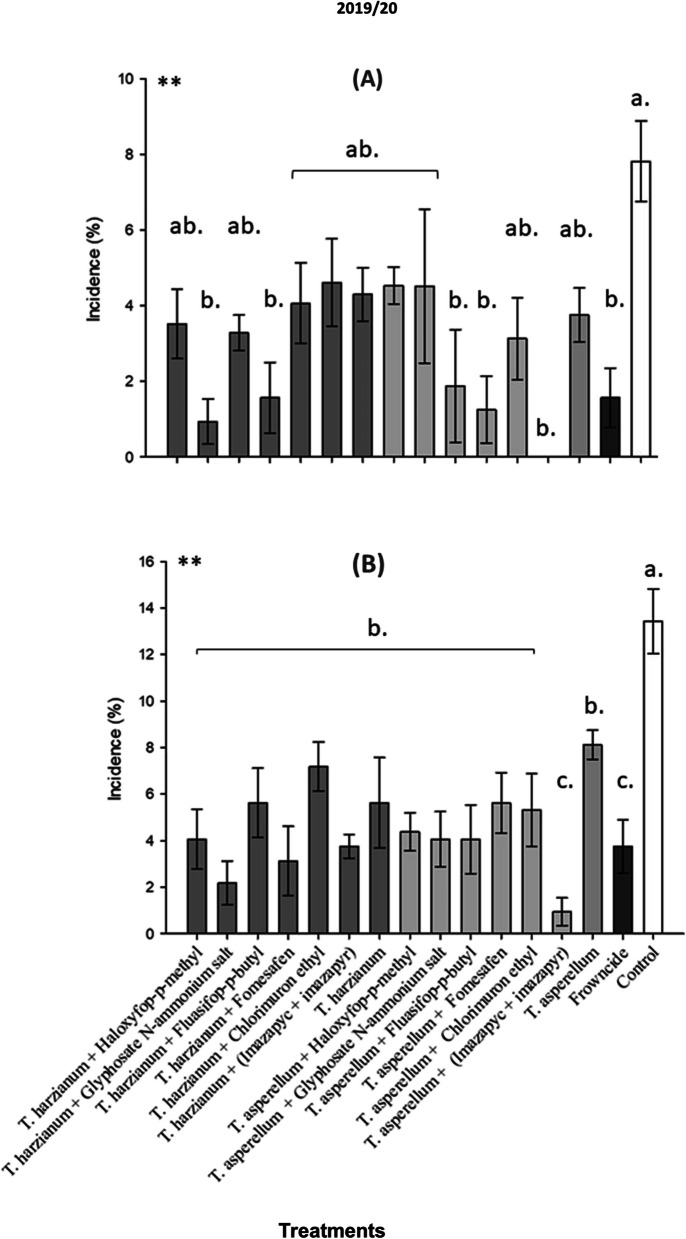
Percentage of white mold (*Sclerotinia sclerotiorum*) incidence in soybean plants according to different treatments with tank mix of herbicides and biological fungicides, under field conditions, 2019- 2020 season. **A** R5.1 soybean growth stage; **B** R5.4 soybean growth stage; season. **C** R6 soybean growth stage. Mean followed by the same letter are not significantly different (*p* < 0.05) according to the Tukey’s HSD test

**Fig. 4:**
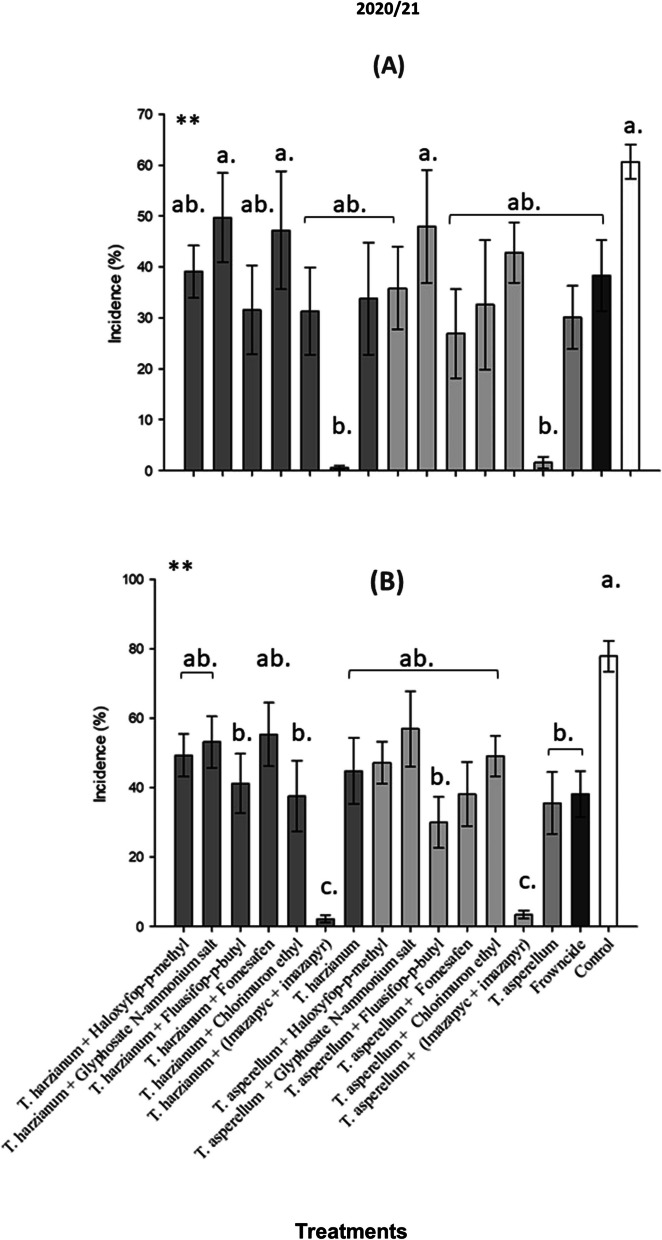
Percentage of white mold (*Sclerotinia sclerotiorum*) incidence in soybean plants according to different treatments with tank mix of herbicides and biological fungicides, under field conditions, 2020- 2021 season. **A** R5.1 soybean growth stage; **B** R5.4 soybean growth stage; season. **C** R6 soybean growth stage. Mean followed by the same letter are not significantly different (*p* < 0.05) according to the Tukey’s HSD test

The bioproducts have been evaluated for the white mold control and plant yield over a two-year field trial (2019/2020) (Fig. 3A & B), and (2020/21) (Fig. 4A & B).

The first white mold incidence assessment for 2019/2020 (1st AV) with an average incidence in the control of approximately 8%, which was lower than the other treatments which ranged from 0.0% to 4.37% (Fig. 3A). The treatments encompassing the application of *T. asperellum* + (Imazapyc + Imazapyr) and *T. harzianum* + Glyphosate Salt Di-ammonium of N showed the lowest incidence of white mold with respectively 0.0% and 0.94% (Fig. 3A). The second evaluation was carried out on February 16th in the 2019/2020 season. In this evaluation, the control of the 19/20 crop experiment obtained an average incidence percentage of 13.43%, differing statistically from the other treatments. The remainder of the treatments did not show statistically significant differences when compared to control (Fig. 3B).

The first white mold incidence assessment for 2020/2021 (1^st^ AV) was carried out on January 5^th^, 2020 with an average incidence in the control of approximately 60% and only *T. harzianum* + (Imazapyc + Imazapyr) and *T. asperellum* + (Imazapyc + Imazapyr) showed significant differences when compared to control (Fig. 4A).

The second evaluation was carried out on February 16^th^, 2020/2021 season. Regarding the 20/21 season, the average incidence of white mold in the control exceeded 80%, being statistically different from the other treatments. Meanwhile, in both treatments that were applied together with the herbicide based on Imazapyc + Imazapyr, the lowest percentage of incidence was recorded with 5.93% for the treatment *T. asperellum* + (Imazapyc + Imazapyr) and 4.68% for the treatment *T. harzianum* + (Imazapyc + Imazapyr), (Fig. 4B).

These results corroborate those obtained in the 19/20 season, where these same treatments with the application of the herbicide based on Imazapyc + Imazapyr resulted in the lowest incidence of white mold in the plants. The occurrence of a lower incidence of white mold in these treatments is correlated with the architecture of the plant and development variables, such as plant height, maturity and lodging. As the soybean cultivars used in the two trials are not tolerant to the herbicide based on Imazapyc + Imazapyr, its phytotoxicity occurred on the cultivars, causing the crop to stunt.

### Yield production

**Fig. 5:**
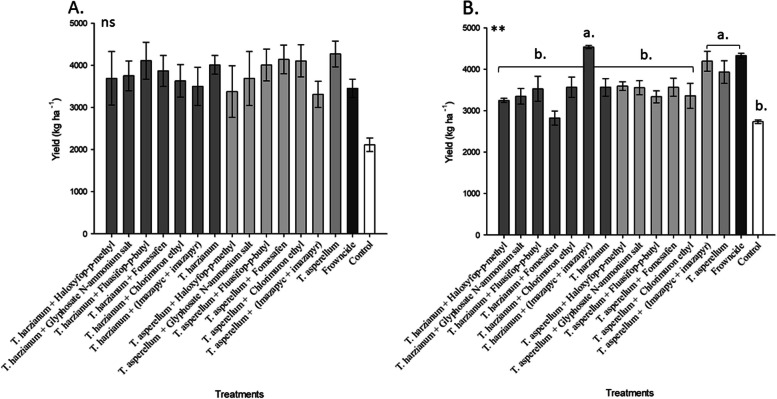
Yield as a function of different treatments with tank mix of herbicides and biological fungicides under field conditions. **A** 2019 season; **B** 2020 season. Mean followed by the same letter are not significantly different (*p* < 0.05) according to the Tukey’s HSD test

Regarding soybean grain yield, (in the 19/20 season no statistical differences were observed between treatments with the application of biological control agents to the control treatment without the application of fungicide (Fig. 5A).

In the 20/21 season, the most productive treatments (*T. harzianum* + (Imazapyc + Imazapyr), Fluazinam, *T. asperellum*, *T. asperellum* + (Imazapyc + Imazapyr), *T. harzianum* and *T. harzianum* + (Imazapyc + Imazapyr) showed significant differences compared to the control, with productivity averages higher than the control and the other treatments (Fig. 5B).

## Discussion

The herbicide Haloxifope-p-methyl, classified as an ACCase inhibitor, exhibited the second highest level of detrimental effects on the biocontrol agent *T. asperellum*. The MGRI of the substance was determined to be 2.84 mm day^−1^, placing it in a lower position compared to Glyphosate. The results of this study suggest that the two herbicides have the most significant influence on the growth of *Trichoderma* spp., with Glyphosate ranking second in relation to impact. In relation to *T. asperellum*, both Fomesafen and Chlorimuron Ethyl herbicides have shown similar efficacy in suppressing the mycelial growth of the fungus. The MGRI of the antagonist was determined to be 6.13 mm per day in both situations (Fig. 2 and Table 2).

Although the fungi do not have a phototrophy mechanism, it was observed that the herbicide based on Fomesafen caused a reduction in the mycelial growth of *T. asperellum*, being statistically similar to Chlorimuron ethyl. According to Malkomes (2000) [31], the additives present in the formulation of pesticides can affect microorganisms and, in certain cases, even modify the effect of the pesticide.

Garcia et al. [32] found that Chlorimuron ethyl at 100 mg L^−1^ significantly reduced mycelial growth in *S. sclerotiorum* isolates from Indianópolis/GO and Jataí/GO. They also tested Fomesafen at the same concentration, resulting in a 72.8% mycelial growth reduction for the Jataí/GO isolate and a 44.0% reduction for the other.

In this study, Chlorimuron ethyl was utilized in conjunction with two herbicides that share similar mechanisms of action.: Haloxifope-p-methyl at a concentration of 311.75 mg L^−1^ and Fluasifope-p-butyl at a concentration of 1,250 mg L^−1^. It is important to note that the concentrations used in this experiment were lower than those employed by the authors and involved different formulations of the herbicides. The herbicides, Fluasifop-p-butyl and Haloxifop-p-methyl, belong to the Aryloxyphenoxypropionic Acid chemical group. These herbicides operate by inhibiting acetyl-coenzyme A carboxylase (ACCase), an enzyme found widely in nature, including in bacteria and fungi. ACCase catalyzes the initial and irreversible step of fatty acid synthesis, a fundamental metabolic pathway in living organisms, converting acetyl co-A into malonyl co-A [33]. Consequently, Fluasifop-p-butyl and Haloxifop-p-methyl herbicides can directly impact microorganisms through this mode of action.

Assessing the potential of herbicides for the control of soil pathogens in bean crops, Lehner et al*.* (2014) [34] found that the active ingredient Imazamox at a concentration of 1,000 mg L^−1^ had the worst performance among herbicides in reducing the colony diameter of fungi, including *S. sclerotiorum*. This active ingredient is part of the chemical group of imidazolinones, whose mechanism of action is the inhibition of acetolactate synthase (ALS). The herbicide Imazapyc + Imazapyr is in the same group as Imidazolinones, so we can see that this chemical group tends not to harm the pathogen.

*Trichoderma* employs multiple mechanisms, such as mycoparasitism, nutrient competition, and the induction of systemic resistance, to help plants resist against *S. sclerotiorum*. *Trichoderma* exhibits mycoparasitic nature by directly engaging in antagonistic interactions with pathogens [35]. It invades and parasitizes the mycelium of the pathogen, consequently hindering its growth and development [36]. In addition, *Trichoderma’*s ability to surpass *S. sclerotiorum* in acquiring nutrients confers an ecological benefit by restricting the resources accessible for the pathogen’s growth. Moreover, *Trichoderma* induces systemic resistance in plants, strengthening their defence systems against subsequent pathogenic invasions [37]. It is essential to comprehend the molecular and biochemical aspects of *Trichoderma* interactions, such as the secretion of antimicrobial peptides and enzymes. This knowledge is crucial for restraining the growth of *S. sclerotiorum*, triggering plant defence mechanisms, and enhancing the effectiveness of herbicides by regulating gene expression to provide a stronger defence against the pathogen [38].

The synergy observed between *Trichoderma* and herbicides, specifically those target certain metabolic pathways of *S. sclerotiorum*, illustrates their interaction [38]. The herbicides increase the efficacy of *Trichoderma* by suppressing the pathogen’s defense mechanisms, rendering it more vulnerable to the biocontrol agent. At the same time, *Trichoderma* enhances the herbicidal effect by offering a durable and biological element for disease control. This combination enhances disease control by concurrently employing biological and chemical strategies to target the pathogen, effectively tackling various stages of its life cycle and minimizing the probability of resistance emergence [39–44].

However, on the other hand, we have the herbicide Clorimuron ethyl, which is inserted in the same mechanism of action, except that of the chemical group of Sulphonylureas, which significantly reduced the mycelial growth of the pathogen and did not allow the formation of sclerotia in the concentration of 100 mg L^−1^.

Corroborating these results, da Costa et al., (2004) [45] pointed out that Glyphosate at a dose of 6.0 L ha^−1^ significantly reduced vegetative growth and sporulation of *M. anisopliae*. Other studies carried out with *B. thuringiensis*, showed results similar to this study, where the Glyphosate-based herbicide inhibited the formation of colonies, and it is not possible to quantify the growth. Thus, the herbicide was classified as incompatible with the entomopathogen because it does not allow the formation of colony forming units (CFU mL^−1^) [45, 46].

While the existing literature does provide some information, there remains a notable gap in understanding. Although there is a degree of consensus regarding compatibilities or incompatibilities, these findings are often based on different evaluation methodologies [47]. It is essential to note that under real-world field conditions, biocontrol agents are exposed to chemical pesticides in the spray solution for a relatively brief period, typically not exceeding 24 h. Subsequently, they must thrive on plants and agricultural residues [48, 49]. Consequently, methodologies that subject antagonists to the chemical pesticide for extended periods, such as seven or more days, may inadvertently lead to an overestimation of pesticide incompatibility with antagonists. This overestimation can paint a scenario of the most adverse conditions concerning time and exposure, frequently resulting in perceptions of incompatibility [25, 50].

Similar results were found by Nelson and Duxbury (2008) [51], who concluded that most of the microorganisms present in soils contained only a functional ALS (Acetolactate synthase) enzyme that is sensitive to the action of ALS-inhibiting herbicides and unable to perform their biocontrol activities against plant soil-borne pathogens. Herbicides based on Haloxifope-p-methyl, Fluasifope-p-butyl, Fomesafen, Chlorimuron ethyl and Imazapyc + Imazapyr in association with the antagonists did not cause their severe inhibition at different times of in vitro exposure, so these herbicides are compatible with the biocontrol agents *T. harzianum* and *T. asperellum*. However, it is believed that in the field, the action of these herbicides is even less damaging to the fungus due to the influence of external factors of the environment, avoiding such intense contact between the chemical and the biological [45], as in the case of glyphosate-based herbicides that are strongly adsorbed on soil particles, becoming inactive [52], or processes such as drift, a gradual decrease in product concentration due to abiotic factors, or even irregular product deposition in the field [53].

These results are significant because this methodology closely simulates real field conditions, in contrast to the previous approach, where the fungus was exposed to the herbicide in a controlled, isolated environment for seven days or longer. In this prolonged exposure scenario, the herbicide affects the fungus on multiple fronts, particularly if there is a tendency for the herbicide to volatilize into the atmosphere, saturating the closed environment within the Petri dish. This phenomenon was more apparent in the 20/21 season, when there was a higher pressure of white mold inoculum and treatments with the application of this herbicide obtained a greater reduction in the incidence of white mold.

The vast majority of compatibility research is carried out using in vitro methodologies or controlled conditions, which are important as a reference. However, the field response may differ considering the dispersion of the active ingredients in the soil and the stability of the biological agent, in addition to the influence of numerous external factors [54].

White mold can develop within a broad temperature range (17–25 ºC). Mild temperatures (17–20 ºC) prompt carpogenic germination and the onset of disease epidemics. At higher temperatures (21–25 ºC) (Fig. S3A), only the myceliogenic phase occurs, which typically causes less damage to the current crop when there is low sclerotia pressure [55]. Notably, in December, for both trials, there was a substantial 300 mm of rainfall (Fig. S3B). This coincided with the crop’s flowering, creating a favorable microclimate for the pathogen, facilitating its infection and subsequent spread within the plant canopy. However, white mold incidence was not high during the 2019/2020 season, while it was considerably higher during the 2020/2021 season. The climatic condition date was shown in Figs. S1A & B and S2A & B. The maximum and minimum temperate mentioned in (Fig. S1A). Notably, in December, for both trials, there was a substantial 300 mm of rainfall (Fig. S2B).

The productivity increases observed in treatments with the isolated application of antagonists may be attributed to the genus *Trichoderma’*s capacity to enhance plant growth, leading to increased crop productivity, increased nutrient absorption, and the induction of resistance to abiotic stresses [56, 57]. It is possible that the herbicides exerted a detrimental effect on these growth-promoting mechanisms of the antagonists, preventing them from reaching their full potential when used in combination with herbicides. The action of these biocontrol agents as growth stimulators is multifaceted and involves interactions with biochemical factors and the production of various enzymes and beneficial compounds. These processes can be influenced by synthetic molecules found in chemical pesticides, such as herbicides [58].

Compact and balanced plants tend to enhance photosynthesis efficiency. According to Liu et al. [59], providing increased light to soybean plants during the early flowering stage results in a higher number of effective pods at the end of the growth cycle, subsequently boosting productivity. During the 2019/2020 season, productivity gains over the control treatment ranged from 19.9 to 36.0 bags ha-1. In the 2020/2021 season, increases ranged from 9.1 to 30.0 bags ha-1. These findings underscore the vital importance of implementing disease control measures; as potential crop damage can significantly impact productivity. Profitability gains relative to the control were calculated using the April 9, 2021 price quotation of 30.96 US$/bag.

## Conclusion

Our research highlights the potential of biological control, specifically the integration of Trichoderma-based solutions, as an effective technique for the holistic management of white mold. The field studies conducted over a period of two years have proven that the herbicides are compatible during a particular time period. This highlights the significance of considering timing when making recommendations for tank mixing. Significantly, when the herbicide imazapique + imazapyr were combined with biocontrol agents, there were notable decreases in the incidence of white mold disease and the generation of conidia. Additionally, there was successful parasitism by *S. sclerotiorum*. The combined effects resulted in a significant increase in crop productivity. Our results highlight the potential benefits of reducing exposure time, which enhances biocontrol viability and performance. This research provides useful information for both practitioners and researchers, promoting a more nuanced and successful approach to managing white mold disease. Future research should extensively investigate and improve compatibility testing procedures, examine other combinations of herbicides and biocontrol agents, and investigate the fundamental molecular mechanisms that drive these interactions in order to enhance integrated pest management systems.

## Supplementary Information

**Supplementary Material 1.**

## Data Availability

All data used in this study are included in this article.
