# Supplementary material for: How can an in vitro incompatibility of Trichoderma-based products and herbicides impact the parasitism and control of white mold (Sclerotinia sclerotiorum (Lib.) De Bary)?
Source: Crop Health. 2024 Apr 9;2(1):5. doi: 10.1007/s44297-024-00024-1 (PMC12825989; doi:10.1007/s44297-024-00024-1)
Supplement: Supplementary file 1 — Supplementary Material 1. [file 44297_2024_24_MOESM1_ESM.docx]

How can an in vitro incompatibility of Trichoderma-based products and herbicides impact the parasitism and control of white mold (*Sclerotinia sclerotiorum* (Lib.) De Bary)?

Lindomar Canuto^1^, Amanda F Faria^2^, Rafaela A Guimaraes^2^, Muhammad Siddique Afridi^2^, Flavio H V Medeiros^2^, Fernanda Carvalho Lopes de Medeiros^1∗^

^1^Department of Agriculture, Universidade Federal de Lavras, Campus Universitario, CP, MG, 3037, 37200900, Lavras, Brazil

^2^Department of Plant Pathology, Universidade Federal de Lavras, Campus Universitario, CP, MG, 3037, 37200900, Lavras, Brazil

Corresponding author

Fernanda C L Medeiros

(Assistant Professor)

Department of Agriculture, Universidade Federal de Lavras, Campus Universitario, CP, MG, 3037, 37200900, Lavras, Brazil

Email: fernandamedeiros@ufla.br

**Figures**

**A**

**B**

**Figure S1**- Climatic conditions during the execution of the experiment in the 2019/2020 harvest.

**A**

**B**

**Figure S2** - Climatic conditions during the execution of the experiment in the 2020/2021 harvest

**Tables**

| **Table S1 -** Description of treatments, concentration of the active ingredient and dose of the commercial product used per hectare. UFLA, Lavas, MG, Brazil 2021. |
| --- |
| \| **Treatment (Herbicides)** \| **Concentration** \| **Dose (C.P)** \| \| --- \| --- \| --- \| \| Control  Haloxyfop-p-methyl \| ---------  124.7 g L^-1^ \| --------  0.5 L ha^-1^ \| \| Glyphosate N Di-ammonium Salt \| 445.0 g L^-1^ \| 6.0 L ha^-1^ \| \| Fluasifop-p-butyl \| 250.0 g L^-1^ \| 1.0 L ha^-1^ \| \| Fomesafem \| 250.0 g L^-1^ \| 1.0 L ha^-1^ \| \| Ethyl Chlorimuron \| 250.0 g kg^-1^ \| 80.0 g ha^-1^ \| \| Imazapique + Imazapir \| 525.0 g kg^-1^ + 175,0 g kg^-1^ \| 150.0 g ha^-1^ \| \| **Biologicals** \| **Recommended concentration** \| **Dose (C.P)** \| \| *T. harzianum* (IBLF 006) \| 1,0 x 10^10^ UFC g^-1^ \| 150,0 g ha^-1^ \| \| *T. asperellum* (BV10) \| 1,0 x 10^10^ UFC g^-1^ \| 150,0 mL ha^-1^ \| |

|  |
| --- |
